# Molecular and Functional Analysis of Trehalose-6-Phosphate Synthase Genes Enhancing Salt Tolerance in *Anoectochilus roxburghii* (Wall.) Lindl.

**DOI:** 10.3390/molecules28135139

**Published:** 2023-06-30

**Authors:** Lin Yang, Luwei Dai, Hangying Zhang, Fuai Sun, Xuchong Tang, Wenqi Feng, Haoqiang Yu, Juncheng Zhang

**Affiliations:** 1 Medical Plant Exploitation and Utilization Engineering Research Center, Sanming University, Sanming 365004, China; yangl@fjsmu.edu.cn (L.Y.); 18159032592@163.com (L.D.); 18005985018@163.com (H.Z.); 2 Maize Research Institute, Sichuan Agricultural University, Chengdu 611130, China; faisun@ucdavis.edu (F.S.); wqfeng93@gmail.com (W.F.); yhq1801@sicau.edu.cn (H.Y.); 3 Bayecao Biotechnology (Sanming) Co., Ltd., Sanming 365004, China; tangxuchong@hqu.edu.cn

**Keywords:** trehalose-6-phosphate synthase, glycometabolism, kinsenoside, salt stress, *A. roxburghii*

## Abstract

Trehalose is a reducing disaccharide, acting as a protectant against various environmental stresses in numerous organisms. In plants, trehalose-6-phosphate synthase (TPS) plays a crucial role in trehalose biosynthesis. *Anoectochilus roxburghii* (Wall.) Lindl. is a prominent species of the *Anoectochilus* genus, widely utilized as a health food. However, the functional analysis of TPS in this species has been limited. In this study, TPS genes were cloned from *A. roxburghii*. The *ArTPS* gene, with an open reading frame spanning 2850 bp, encodes 950 amino acids. Comparative and bioinformatics analysis revealed that the homology was presented between the ArTPS protein and TPSs from other plant species. The ORF sequence was utilized to construct a prokaryotic expression vector, Pet28a-ArTPS, which was then transformed into *Escherichia coli*. The resulting transformants displayed a significant increase in salt tolerance under the stress conditions of 300 mmol/L NaCl. Quantitative RT-PCR analysis demonstrated that the expression of *ArTPS* genes responded to NaCl stress. The accumulation of G6P was upregulated, whereas the content of T6P exhibited an opposite expression trend. The glycometabolism products, including trehalose, exhibited notable changes under NaCl stress, although their variations may differ in response to stimulation. The content of kinsenoside, a characteristic product of *A. roxburghii*, was significantly upregulated under NaCl stress. These results suggest that the *ArTPS* genes function in response to NaCl stimulation and play a key role in polysaccharide and glycoside metabolism in *Anoectochilus*. This study provides new insights into the engineering modification of the health food *A. roxburghii* to enhance the medicinal activity of its ingredients.

## 1. Introduction

*Anoectochilus roxburghii* (Wall.) Lindl., an orchidaceae species, has a number of medicinal components that are essential for treating cancer and allergies [1,2,3,4]. Because to its protected status, the wild *A. roxburghii* is frequently exploited in artificial cultivation or tissue culture for quick multiplication [5,6,7]. However, unlike wild plants, cultivated *A. roxburghii* exhibits distinct accumulation patterns of medicinal components, mainly including polysaccharides and the glycoside derivative kinsenoside, primarily through secondary metabolites [5,8]. In the artificial cultivation or tissue culture of *A. roxburghii*, it is crucial to promote the accumulation of glycometabolites and glycoside derivatives.

Trehalose is a non-reducing disaccharide that is widely distributed in both plants and microbes [9]. While it is known to be present in specific drought-tolerant plants, it has also been identified in common plants such as rice and *Arabidopsis* [10,11]. However, its content in these plants is typically very low [12,13]. In plants, trehalose synthesis is first catalyzed by trehalose-6-phosphate synthase (TPS), which transfers glucose from UDP-glucose to glucose-6-phosphate (G6P) to produce trehalose-6-phosphate (T6P). Then, T6P was dephosphorylated by trehalose-6-phosphate phosphatase (TPP) to produce trehalose [14,15]. Among these genes, the *TPP* gene can be substituted by the *TPS* gene, and TPS plays a crucial role in regulating the rate of biosynthesis for T6P and trehalose metabolites. These components are vital for a wide range of physiological and biochemical processes. [14,16].

In previous studies, the *TPS* gene was cloned and identified as *AtTPS1* from cDNA libraries of Arabidopsis plants [17]. As research has progressed, TPS genes have been successfully cloned in various other plant species, such as *Medicago truncatula*, *Dioscorea esculenta*, and *Tamarix hispida* [15,18,19]. TPS has garnered significant attention, particularly due to its involvement in stress resistance mechanisms. The TPS gene has been observed to be upregulated and expressed in response to various stress conditions such as cold, salt, drought, and mannitol stresses in ginkgo plants [20]. The expression of the *TPS* gene was elevated under cold stress, and this gene may be related to the cold tolerance mechanism in tea trees [21]. The *AtTPS5*-dependent trehalose metabolism mediates basal defense responses [22]. In maize, the *TPS* gene was involved in the accumulation of trehalose and induced the expression of related genes in the early stages of salt and cold stress; in the late stages of stress, the *TPS* gene was upregulated and accumulated T6P, which opened other stress response pathways through the regulation of polysaccharide metabolism [14,23]. Thus, it was clear that *TPS* gene expression not only affected plant stress resistance but also regulated glycometabolism and glycoside through T6P.

In the present study, the TPS genes were amplified from A. roxburghii. After bioinformatics analysis, the expression of ArTPS genes in response to precursor substance NaCl stress was detected by real-time quantitative PCR (RT-qPCR). The transformation of the ArTPS genes into E. coli could increase tolerance to salt and cold. And the polysaccharides quantification and kinsenoside measurement of A.roxburghii were detected under the NaCl stress. These results demonstrate that ArTPS genes play an important role in glycometabolism and glycoside biosynthesis in Anoectochilus.

## 2. Results

### 2.1. Open Reading Frame and Putative Proteins

The TPS gene sequences from *A. roxburghii* were obtained through RNA-seq analysis. To amplify segments longer than 2000 and 3000 base pairs, appropriate primers were designed based on the provided information (Figure S1). The ArTPS gene consists of a single open reading frame spanning 2850 bp, encoding 950 amino acids. It has a molecular weight of 106.78 kDa, isoelectric point values of 6.81, and GRAVY values of −0.345. The molecular formula of ArTPS is C_4737_H_7424_N_1350_O_1410_S_29_. The secondary structure of ArTPS comprises 41.47% α-helices, 13.32% extended strands, and 38.11% random coils. These structural elements are present in the three-dimensional model of ArTPS (Figure S2). Notably, the characteristics of ArTPS, PeTPS, and DcTPS were found to be comparable.

### 2.2. Multiple Sequence Alignment and Phylogenetic Analysis

**Figure 1:**
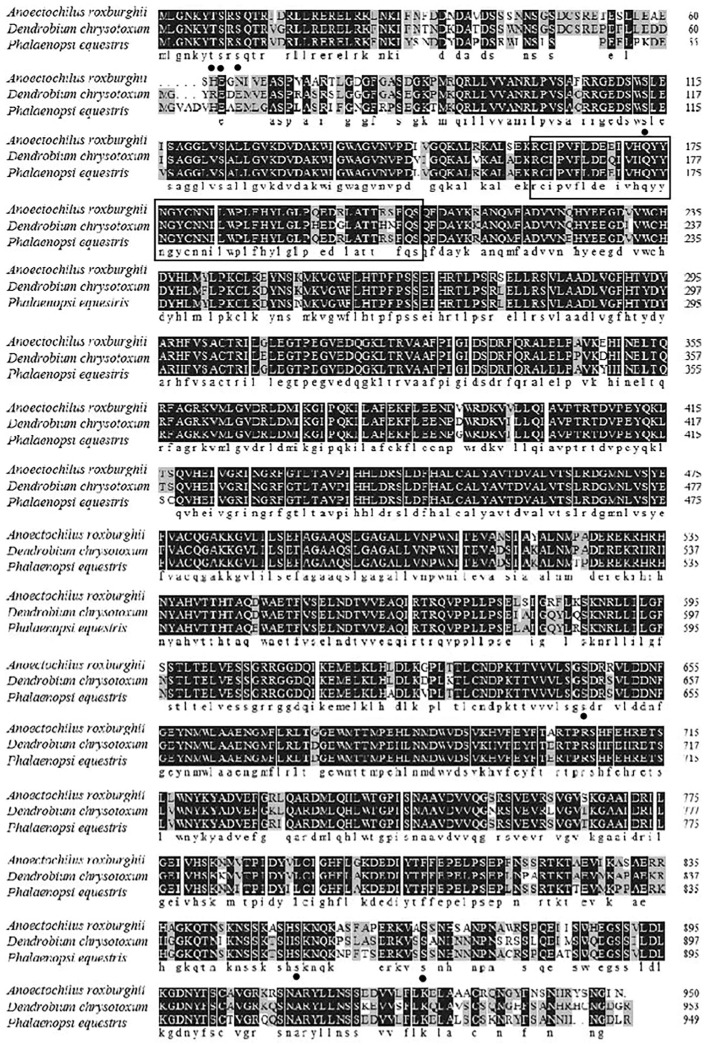
The structural functional domain of the *TPS* genes among *A. roxburghii*, *Dendrobium chrysotoxum,* and *Phalaenopsis equestris*. Identical and conserved amino acid residues are denoted by black (100%), gray (66.6%), and white (0%) backgrounds, respectively. The box with solid line represents the conserved motif, while the black dots represent the phosphorylation sites.

**Figure 2:**
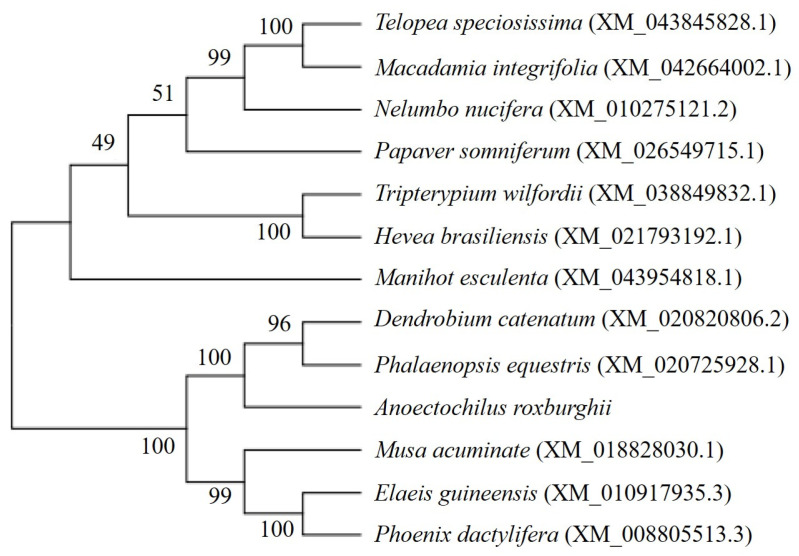
Phylogenetic tree among putative TPS protein of *A. roxburghii* and deposited functional TPS proteins of other plants.

A glycosyltransferase conserved domain and a HAD-like conserved domain were found in position 58-558 and 612-848, respectively. Meanwhile, the phosphorylation sites of T7, S8, S10, S113, S646, S852, and S868 were found in the ArTPS protein, respectively. Moreover, the conserved motif R160-S206 was found in multiple sequence alignment from TPS (Figure 1). Multiple alignment and phylogenetic analysis showed that the putative ArTPS proteins were clustered into the same sub-group with the deposited functional TPS proteins of *Dendrobium huoshanense* and *Phalaenopsis equestris* (Figure 1 and Figure 2), indicating that TPS from *A. roxburghii* is a member of the TPS family. 

### 2.3. Overexpression of TPS Gene

**Figure 3:**
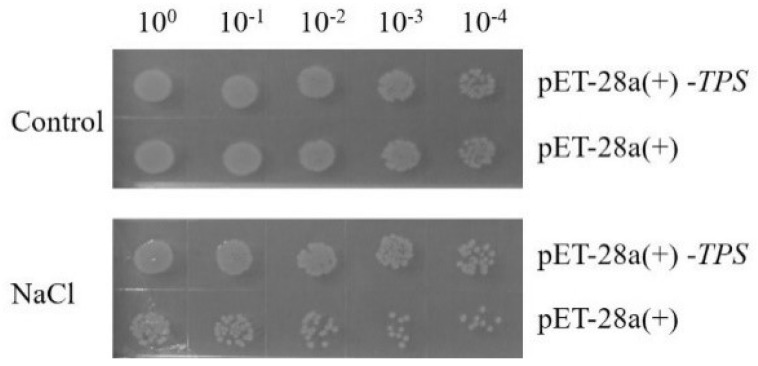
Growth status of the *E. coli* colonies transformed by pET−28a(+) and pET−28a(+)−*TPS* under the NaCl of 300 mmol L^−1^.

A noticeable reduction in colony numbers on the LB agar plates was observed as the dilution fold increased. Furthermore, clear distinctions were observed between the lines transformed with the loaded vector pET−28a(+)−*TPS* and the empty vector pET−28a(+). Under the stress of 300 mmol L^−1^ NaCl, the E. coli colonies transformed with pET−28a(+)−*TPS* exhibited significantly higher tolerance to salt stress compared to those transformed with pET−28a(+) (Figure 3).

### 2.4. Relative Expression Level under Induction

**Figure 4:**
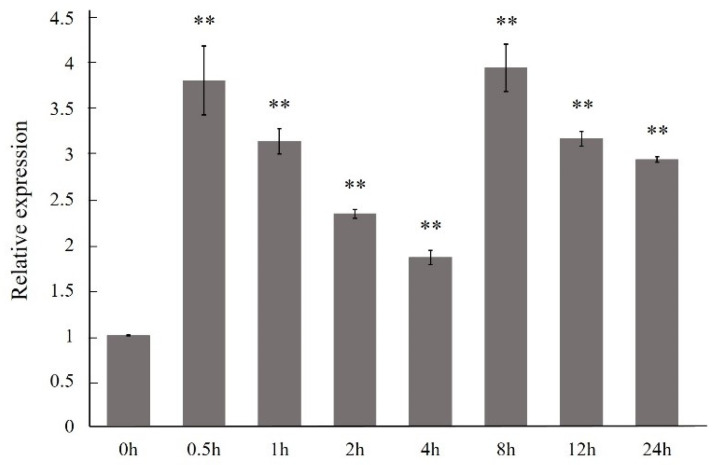
Relative expression levels of *ArTPS* genes under the NaCl stress in *A. roxburghii*. The double asterisk (**) stands for significance with the control at 0.01 levels.

The expressions of the *ArTPS* gene were significantly upregulated in *A. roxburghii* and there was a peak value at 0.5 h (3.81 times) in response to NaCl stress at the beginning. Then, the expressions plummeted and reached its valley at 4 h, only half or even less at 0.5 h. And the expressions were upregulated, and there was a peak value at 8 h (3.95 times). The *ArTPS* genes were continually downregulated at 12 h and 24 h (Figure 4). This result indicated the strong expression response of these *ArTPS* genes.

### 2.5. G6P and T6P Accumulation under Inducion

**Figure 5:**
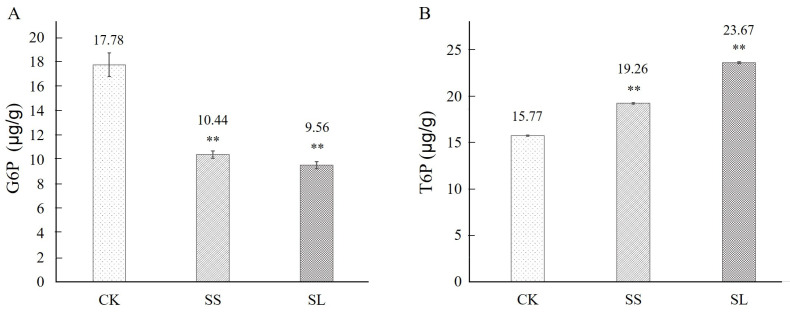
The contents of G6P and T6P in *A. roxburghii* under non-inducing condition and NaCl stress. A: The contents of G6P in *A. roxburghii* under non-inducing condition and NaCl stress; B: The contents of T6P in *A. roxburghii* under non-inducing condition and NaCl stress. CK stands for non-inducing; SS stands for NaCl stress after 4 d; SL stands for NaCl stress after 16 d. The double asterisk (**) stands for significance with the control at *p* < 0.01 levels.

Under non-inducing conditions (the 0 h control), the accumulation of G6P and T6P of *A. roxburghii* was 17.78 μg/g and 15.77 μg/g from *A. roxburghii*, respectively (Figure 5). In response to NaCl induction, the accumulation of G6P was both decreased by 41.28% and 46.24% after 4 d and 16 d, respectively (Figure 5A), indicating its expression suppressed. On the contrary, the accumulation of T6P was 1.22 and 1.50 times (19.26 μg/g and 23.67 μg/g) of that after 4 d and 16 d by NaCl stress (Figure 5B). 

### 2.6. Glycometabolism and Kinsenoside Accumulation under Induction

**Figure 6:**
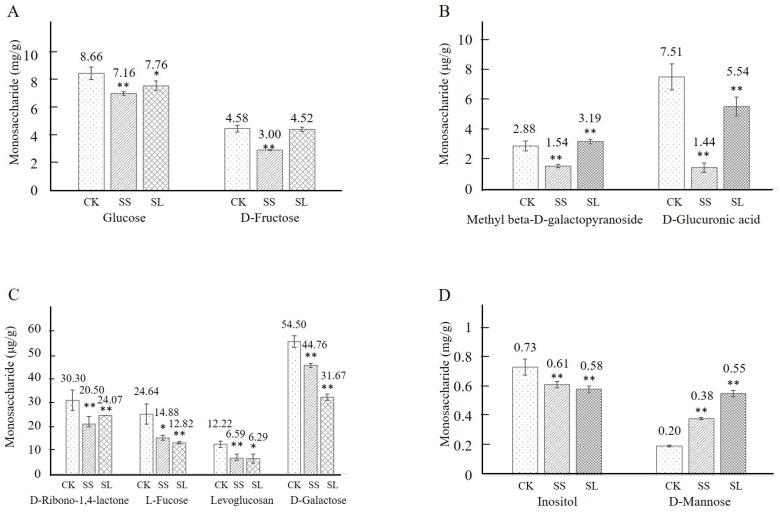
The monosaccharide contents in *A. roxburghii* under non-inducing conditions and NaCl stress. (**A**) The contents of Glucose and D-Fructose in *A. roxburghii* under non-inducing condition and NaCl stress; (**B**) The contents of Methyl beta-D-galactopyranoside and D-Glucuronic acid in *A. roxburghii* under non-inducing condition and NaCl stress; (**C**) The contents of D-Ribono-1,4-lactone, L-Fucose, Levoglucosan and D-Galactose in *A. roxburghii* under non-inducing condition and NaCl stress; (**D**) The contents of Inositol and D-Glucuronic acid and D-Mannose in *A. roxburghii* under non-inducing condition and NaCl stress. CK stands for non-inducing; SS stands for NaCl stress after 4 d; SL stands for NaCl stress after 16 d. The asterisk (*) and double asterisk (**) stand for significance, with the control at *p* < 0.05 and *p* < 0.01 levels, respectively.

The accumulation of monosaccharide from Glucose, D-Fructose, Methyl beta-D-galactopyranoside, D-Glucuronic acid, and D-Ribono-1,4-lactone was decreased during the first four days of the induction but rebounded after that with different ranges between different products (Figure 6A–C). The content of monosaccharide from L-Fucose, Levoglucosan, Inositol, and D-Galactose showed a continuous decrease over the 16 days measured (Figure 6C-D). But the accumulation of D-Mannose was increased continually (Figure 6D). 

**Figure 7:**
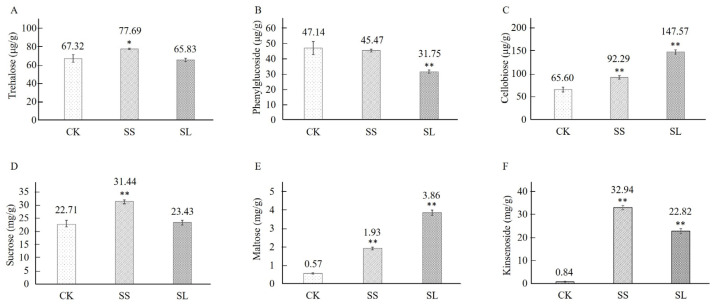
The contents of monosaccharide in *A. roxburghii* under non-inducing condition and NaCl stress. (**A**) The contents of trehalose in *A. roxburghii* under non-inducing condition and NaCl stress; (**B**) The contents of phenylglucoside in *A. roxburghii* under non-inducing condition and NaCl stress; (**C**) The contents of maltose in *A. roxburghii* under non-inducing condition and NaCl stress; (**D**) The contents of sucrose in *A. roxburghii* under non-inducing condition and NaCl stress; (**E**) The contents of cellobiose in *A. roxburghii* under non-inducing condition and NaCl stress; (**F**) The contents of cellobiose in *A. roxburghii* under non-inducing condition and NaCl stress. CK stands for non-inducing; SS stands for NaCl stress after 4 d; SL stands for NaCl stress after 16 d. The asterisk (*) and double asterisk (**) stand for significance with the control at *p* < 0.05 and *p* < 0.01 levels, respectively.

**Figure 8:**
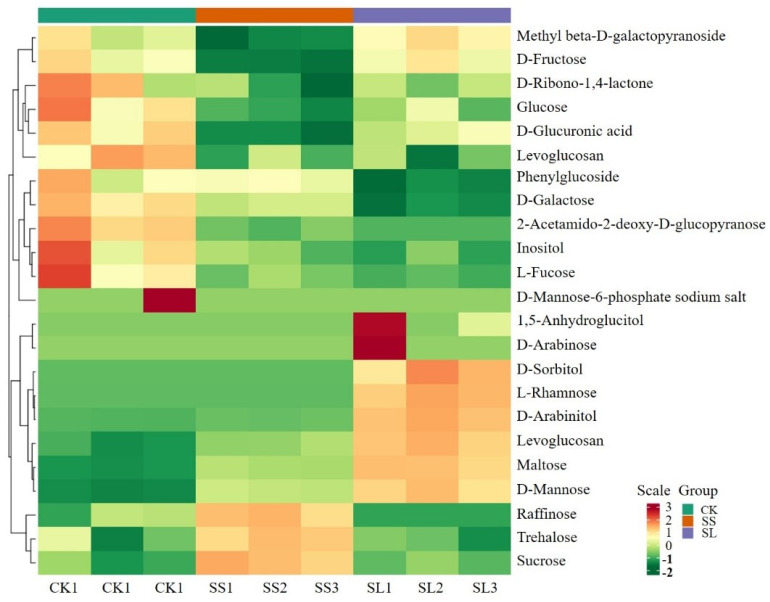
The heat map of glycometabolism content clustering in *A. roxburghii* under non-inducing condition and NaCl stress. CK stands for non-inducing, SS stands for NaCl stress after 4 d, SL stands for NaCl stress after 16 d; red stands for high relative expression, green stands for low relative expression, yellow stands for medium relative expression; the right side indicates the sorting of glycometabolism clusters.

The trehalose content was 67.32 μg/g under 0 h control from *A. roxburghii*. In NaCl induction, the accumulation of trehalose in *A. roxburghii* was increased and reached their peak values (1.15 times of the 0 d control) on the fourth day. The difference of trehalose content was not significant between the 0 h control and the 16th day (Figure 7A). In NaCl induction, the expression of the phenylglucoside were continually downregulated from 47.14 μg/g to 31.75 μg/g (Figure 7B). The accumulation of maltose and cellobiose was significantly lower in the untreated than the salt-treatment model on the 4th and 16th day (Figure 7C,E). The sucrose content was continually upregulated and reached about 1.5 times (31.44 mg/g) of the 0 d control at the fourth day (Figure 7D). In contrast, the downregulated expression reached the same level of the 0 d control at 16 d of the NaCl induction in *A. roxburghii* (Figure 7D). The content of sucrose exhibited a similar trend of change as that of trehalose. (Figure 7D). Using the heat map, the glycometabolism content was clustered in *A. roxburghii* under non-inducing conditions and NaCl stress (Figure 8).

The accumulation of kinsenoside was 0.84 mg/g under 0 h control from *A. roxburghii*. The contents were increased sharply under salt induction, peaking at 32.94 mg/g on the 4th day, and then decreasing to 22.82 mg/g on the 16th day, while the upregulated expression reached 39.21 and 27.17 times of the 0 h control (Figure 7F).

## 3. Discussion

Trehalose metabolism is involved in the growth, development, and abiotic stress response in higher plants [24,25]. TPS is an essential enzyme in the trehalose biosynthetic pathway [26,27]. The *TPS* gene also plays a vital role in abiotic stress tolerance in plants [28,29,30,31,32]. In this study, the *TPS* gene was identified from *A. roxburghii* cDNA. The study presented the homology of the open reading frame (ORF) and putative protein sequences with the deposited functional TPS proteins of *Dendrobium chrysotoxum* as well as *Phalaenopsis equestris*. The TPS proteins contained both a glycosyltransferase conserved domain, a HAD-like conserved domain, and six phosphorylation sites, indicating that the TPS protein is highly conserved and the structures of the two domains might be essential for TPS functions in different plants. This characteristic was consistent with previous studies in *Arabidopsis*, rice, and Populus [19,33,34]. 

Expression level analyses of NaCl stress would help to better understand and predict gene function. The qRT-PCR results show that the expression of the *ArTPS* gene was significantly upregulated after NaCl treatments. Our results were consistent with research in *Medicago truncatula*, *Dioscorea esculenta*, and *Tamarix hispida* [15,19]. The *TPS* gene of *A. roxburghii* could be endowed with the same TPP and TPS catalytic activity as the maize. The *TPS* gene was upregulated in the early stage, and one of its functions could be to accumulate trehalose with the *TPP* gene, thus inducing the expression of downstream stress response related genes. The upregulation of the *TPS* gene expression in the later stage was regulated glycometabolism and glycoside through T6P, which was confirmed by the content of T6P and trehalose [14]. The accumulation of G6P and T6P, as the substrate and product of TPS catalysis, mainly showed opposite expression trends; however, the content of trehalose was short-changed after salt treatments, indicating that the ArTPS may be involved in increasing the T6P content in salt stress responses; while the accumulation of trehalose was increasedto counteract stress for the short-term, it could be maintained at a certain level for the long-term [23]. The other products from the glycometabolism were significantly changed under the NaCl stress, while its variation may differ in responses to stimulation. The content of kinsenoside was sharply upregulated, increasing T6P to regulate the polysaccharide and glycoside metabolism [15,23]. 

The overexpression of the ArTPS gene in E. coli demonstrated higher suitability compared to the control group when subjected to a stress condition of NaCl of 300 mmol L^−1^ stress. *TPS* genes play important roles in plant growth, development, and response to biotic and abiotic stresses [26,27]. *AtTPS1* overexpression can enhance drought resistance in *A. thaliana* [35]. Overexpressing the gene encoding the bifunctional fusion of *TPS* and *TPP* genes from *Escherichia coli* in transgenic tomato plants improved drought and salt resistance and photosynthetic rates [36].

## 4. Materials and Methods

### 4.1. Sample Preparation

The *A. roxburghii* seedlings were surface-sterilized with 10% NaClO for 5 min. Then, they were plated onto MS media in a chamber with a 12 h light/12 h dark photoperiod at 28 °C and 60–80% relative humidity. The Hoagland nutrition solution was used to transplant the 4-month-old seedlings into an aquaculture plastic mesh grid. For stress conditions, 100 nm/L of NaCl was added to the nutritional solution on the sixth day. 

Three biological replicates of the seedlings were collected at time points of 0 h (control), 0.5 h, 1 h, 2 h, 4 h, 8 h, 12 h, 24 h (1 d), 2 d, 4 d, 8 d, 16 d, and 32 d of treatment with two groups. The seedlings of 0 to 12 h were immediately ground in liquid nitrogen for total RNA extraction by a Qiagen RNeasy plant mini kit (Qiagen, China), and reverse-transcribed to cDNA with a Takara PrimeScript RT reagent Kit (TakaRa, China), respectively.

### 4.2. Cloning of the TPS Gene

The forward and reverse primer (5′-ATGCTGGGAAATAAGTACACGAG-3′/5′- TCAAATACCATTGGAATAGCGGTGG-3′) were designed to amplify the *ArTPS* gene open reading frame (ORF) from cDNA based on RNA-seq annotation. PrimeStar HS DNA Polymerase (TakaRa, Japan) was used to carry out the PCR amplification along with the proofreading process. The temperature cycle consisted of 38 cycles of 95 °C for 2 min, 98 °C for 30 s, 56 °C for 30 s, 72 °C for 150 s, and 72 °C for 5 min. The amplified product was purified using the Universal DNA Purification Kit (Tiangen, China), dATP was added to the sequence tails using the TaKaRa TaqTM (TakaRa, China), the amplified product was cloned into the pMD19-T vector (TakaRa, China), and Shanghai Sangon Biotech Co., Ltd. (China) sequenced the product.

### 4.3. Bioinformatic Analysis

The *ArTPS* gene sequencing was aligned for gene structure using NCBI blast (http://www.ncbi.nl-m.nih.gov accessed on 15 April 2023), and the same sequence was used for the analysis of physical and chemical properties, secondary structure, functional domains, and genetic structure of the putative proteins by using ProtParam (http://web.expasy.org/protparam accessed on 15 April 2023), GOR IV (http://npsa-pbil.ibcp.fr/cgibin/npsa_automat.pl?page=npsa_gor4.html accessed on 15 April 2023), TMHMM Server v. 2.0 (http://www.cbs.dtu.dk/services/TMHMM-2.0/ accessed on 15 April 2023), and SWISS-MODEL (https://swissmodel.expasy.org/ accessed on 15 April 2023) software or databases, respectively. The phylogenetic analysis was studied between the putative proteins of ArTPS and the TPS amino acid sequences published in the NCBI database using MEGA7.0 software (https://www.megasoftware.net/ accessed on 15 April 2023). The Poisson correction method was used to calculate the evolutionary distances.

### 4.4. Vector Construction

A pair of specific primers (5′- cgggatccATGCTGGGAAATAAGTACACGAG -3′/5′- ccctcgagTCAAATACCATTGGAATAGCGGTGG -3′) was designed with the introduction of the recognition sites (the lower-case bases in the bracket), and used to amplify the ORF of *ArTPS* without termination codon for homologous recombination by using Prime STAR HS DNA Polymerase (TaKaRa, Japan). The product was inserted into the expression vector pET-28a(+) using CloneExpress One Step Cloning Kit (Vazyme, China), to generate a set of expression vectors bearing fusion genes between the *ArTPS* genes (Figure S3).

### 4.5. Protokaryotic Expression

pET-28a(+)-TPS was transformed into *E.coli* strain Rosetta (DE3) with empty prokaryotic pET-28a(+) as negative control and screened on a Luria–Bertani (LB) agar plate containing 50 mg L^−1^ ampicillin at 37 °C overnight. After confirmation by bacterial PCR and double digestion of the extracted plasmids with *BamH* I/*Xho* I, the positive single colonies were cultured in LB liquid medium containing 50 mg L^−1^ ampicillin at 37 °C. After 4 h, until OD600 = 0.6, the cultures were added with isopropyl β- D -thiogalactopyranoside (IPTG) to a final concentration of 1 mmol L^−1^, and induced at 37 °C for 4 h. Then, each culture was adjusted to OD600 = 0.6 with the ampicillin LB liquid medium. Referring to He et al. (2012) and Udawat et al. (2014), each of the cultures was diluted by ten-fold serial to 1:104 with the ampicillin in LB liquid medium [37,38]. Two microliters of each dilution were spotted on the ampicillin LB agar plates containing NaCl of 300 mmol L^−1^. After incubation for 16 h, the growth status of the colonies was observed.

### 4.6. RT-qPCR

A pair of precise primers (5′- GCATGCCAAGGTGCAAAGAA-3′/5′- GCCTATGTCGCTTCTCCCTC-3′) was created to amplify a 172 bp segment of the *ArTPS* gene. A second set of precise primers (5′-CGGGCATTCACGAGACCAC-3′/5′-AATAGACCCTCCAATCCAGACACT-3′) was created to amplify a 221 bp *Actin2* gene fragment that was used as an internal reference [39]. In the Bio-Rad iCycler iQ5 RT-qPCR System, the amplification reaction was carried out using SsoFast EvaGreen Supermix (Bio-Rad, USA). The two-step RT-qPCR temperature cycle was as follows: 39 cycles of 95 °C for 5 s, followed by 30 s at 56 °C. So that a melting curve could be constructed and utilized to distinguish between specific and non-specific amplicons, the temperature was then raised by 0.5 °C/s to 95 °C. The internal reference and *TPS* gene expression were normalized using the 2^−ΔΔCT^ method of the CFX Manger^TM^ software version 2.0 (Bio-Rad, USA) [40]. The IBM-SPSS software (http://www-01.ibm.com/software/analytics/spss/ accessed on 15 April 2023) was used to evaluate the statistics. 

### 4.7. G6P, T6P, and Glycometabolism Quantification

The seedling sample from 0 d, 4 d, and 16 d were dried at 55 °C, ground to fine powder, with 50 mg powder extracted in 500 mL 70% CH_3_OH. The extracts were through protein precipitation plate (MPPT9601A, Biocomma Limited). Two microliters of each of the filtrates were loaded into UPLC (Waters ACQUITY H-ClassD) equipped with ACQUITY UPLC BEH Amide column (1.7 μm, 2.1 × 100 nm) and MS/MS (QTRAP^®^ 6500+). Gradient elution was conducted with 5% mobile phase A (H_2_O with 10 mM CH_3_COONH_4_ and 0.3% NH_3_·H_2_O) and 95% mobile phase B (CH_3_CN) for 1.2 min, 30% mobile phase A and 70% mobile phase B for 8 min, 50% mobile phase A and 50% mobile phase B for 2 min, and 5% mobile phase A and 95% mobile phase B for 4 min at a flow rate of 1 mL/min and column temperature of 30 °C. And the MS/MS conditions included 550 °C of Elektrospray-Ionenquelle, 5500 V/−4500 V of the mass spectrometry voltage in positive/negative ion mode, and 35 psi Curtain Gas. Each ion pair was scanned through declustering potential and collision energy.

### 4.8. Kinsenoside Measurement

The seedling sample from 0 d, 4 dm and 16 d were dried at 55 °C, ground to fine powder, with 0.5 g powder extracted in 10 mL CH_3_OH in an ultrasonic at 25 °C for 30 min. The extracts were filtered through a 0.22 um filter membrane. Ten microliters of each filtrates were loaded into HPLC (Agilent 1260) equipped with Brownlee SPP C18 column (E-Merck, 2.7 μm, 4.6 × 150 nm). The elution was conducted with 10% CH_3_OH, 5% CH_3_CN, and 85% H_2_O as mobile phase at a flow rate of 1 mL/min and column temperature of 30 °C. The kinsenoside constituents were monitored at 215 nm and identified by comparison of retention time with their authentic samples. Their relative contents were quantified and expressed as percentage peak area.

## 5. Conclusions

The open reading frame sequences of the *TPS* genes were obtained from *A. roxburghii*. After bioinformatics analysis, the homology of the TPS proteins and conserved domains was found in their orthologs in related species. The overexpression of the *ArTPS* gene in *E. coli* showed higher tolerance to salt stress. Meanwhile, the relative expressions of the *TPS* genes were significantly higher under the NaCl stress. *ArTPS* gene expression not only affected plant stress resistance but also regulated the accumulation of glycometabolites and kinsenoside which is the characteristic substances of *A. roxburghii* through T6P.

## Data Availability

The data will be available on request.

## Supplementary Materials

The following supporting information can be downloaded at: https://www.mdpi.com/article/10.3390/molecules28135139/s1, Figure S1: The fragments of the *TPS* gene from cDNA in *A. roxburghii*. Figure S2: Predicted three-dimensional models of the putative proteins of the *TPS* genes between (A) *A.roxburghii*, (B) *D. chrysotoxum,* and (C) *P. equestris*. Figure S3: The structure of vector pET-28a(+)-*TPS*.
