# Supplementary material for: Molecular and Functional Analysis of Trehalose-6-Phosphate Synthase Genes Enhancing Salt Tolerance in Anoectochilus roxburghii (Wall.) Lindl
Source: Molecules. 2023 Jun 30;28(13):5139. doi: 10.3390/molecules28135139 (PMC10343327; doi:10.3390/molecules28135139)
Supplement: Supplementary file 1 [file molecules-28-05139-s001.zip › molecules-2450454-supplementary.pdf]

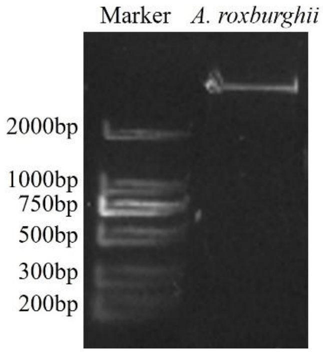

Figure S1. The fragments of the *TPS* gene from cDNA in *A. roxburghii*.

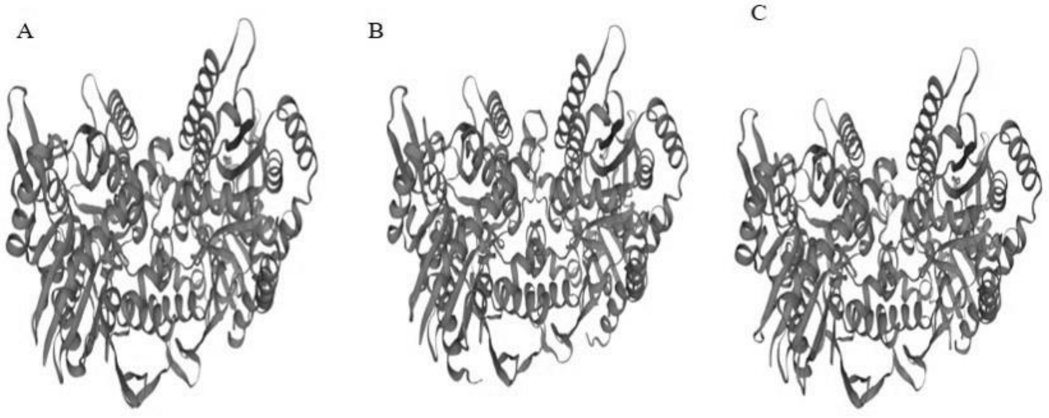

Figure S2. Predicted three-dimensional models of the putative proteins of the *TPS* genes between (**A**) *A.roxburghii*, (**B**) *D. chrysotoxum* and (**C**) *P. equestris*.

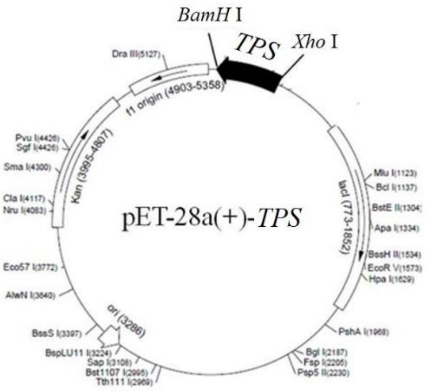

Figure S3. The structure of vector pET-28a(+)-*TPS*.
